# The dynamics of functional classes of plant genes in rediploidized ancient polyploids

**DOI:** 10.1186/1471-2105-14-S15-S19

**Published:** 2013-10-15

**Authors:** Eric CH Chen, Carlos Fernando Buen Abad Najar, Chunfang Zheng, Alex Brandts, Eric Lyons, Haibao Tang, Lorenzo Carretero-Paulet, Victor A Albert, David Sankoff

**Affiliations:** 1 Department of Biology, University of Ottawa, 30 Marie-Curie, Ottawa, Canada, K1N 6N5; 2 Facultad de Ciencias, Universidad Nacional Autónoma de México, Avenida Universidad 3000, Distrito Federal, México; 3 Department of Mathematics and Statistics, University of Ottawa, 585 King Edward Avenue, Ottawa, Canada, K1N 6N5; 4 School of Plant Sciences, iPlant Collaborative Bio5 Institute, University of Arizona, 1657 E Helen St, Tucson, AZ 85745, USA; 5 J Craig Venter Institute, 9704 Medical Center Dr, 20850 Rockville, MD, USA; 6 Department of Biological Sciences, University at Buffalo, Buffalo, New York 14260, USA

## Abstract

**Background:**

To understand the particular evolutionary patterns of plant genomes, there is a need to systematically survey the fate of the subgenomes of polyploids fixed as whole genome duplicates, including patterns of retention of duplicate, triplicate, etc. genes.

**Results**

We measure the simultaneous dynamics of duplicate orthologous gene loss in rosids, in asterids, and in monocots, as influenced by biological functional class. This pan-angiosperm view confirms common tendencies and consistency through time for both ancient and more recent whole genome polyploidization events.

**Conclusions**

The gene loss analysis represents an assessment of post-polyploidization evolution, at the level of individual gene families within and across sister genomes. Functional analysis confirms universal trends previously reported for more recent plant polyploidy events: genes involved with regulation and responses were retained in multiple copies, while genes involved with metabolic and catalytic processes tended to lose copies, across all three groups of plants.

## Introduction

In whole genome duplication (WGD) or triplication, the entire gene and chromosome structure of a genome undergoes polyploidization followed by rediploidization of the new larger genome and fractionation, or homeologous gene loss, of many or most of the duplicate genes. The doubling or tripling of all the gene contents of a genome has been hypothesized as an important source of gene innovations and the radiation of species. The inherent variability in these processes may create a variety of beneficial phenotypes similar to those seen due to heterosis [1], and the increase in the diversity of genetic elements may help drive long-term morphological complexity and adaptation to new environments [2].

Studies in a variety of organisms have provided evidence that gene retention during fractionation may differ among functional categories. This has given rise to a number of explanatory models, particularly a reformulation of the classical genetic Gene Balance Hypothesis [3], all of which attempt to explain functional bias in gene retention after WGD.

**Figure 1:**
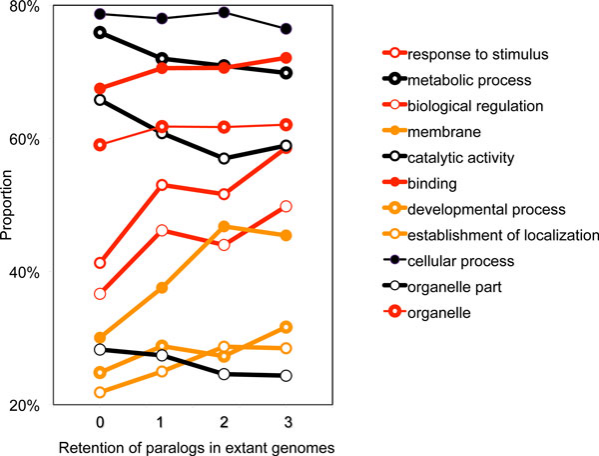
**Proportion of genes with various numbers of extant copies annotated with given high-level functional categorizations**. x-axis: number of genomes (out of three: peach, cacao and grape) with more than one copy of a given gene. Score 0 indicates all three genomes contain exactly one copy of the gene; 1 indicates that one of the genomes has two or three copies, 2 means that two of the genomes have two or three copies and 3 means that all three genomes have two or three copies. y-axis: proportion of these gene sets with the indicated annotation. Positive slope indicates fractionation resistance, negative slope indicates fractionation-prone genes. Thick lines indicate statistical significance of non-zero slope.

In a previous study [4], we proposed a comparative genome-wide analysis of the descendants of triplicated genes in the ancestor of the core eudicots, focusing on three rosid plants that have not undergone any subsequent whole genome duplication: peach (*Prunus persica*) [5], cacao (*Theobroma cacao*) [6], and grape (*Vitis vinifera*) [7]. We asked whether the genes that have been retained in three or two paralogous copies could be seen to be enriched for certain functional categories. These results, updated to reflect the current study, are illustrated in Figure 1.

These results, such as the relatively rapid fractionation of genes labelled "metabolic", and the resistance to fractionation of those labelled "biological regulation", confirmed various accounts in the literature [8-11].

This work raised new questions. The first is to what extent the patterns in Figure 1 are specific to rosids. Which functional categories have the same tendencies in other flowering plant groups? The second concern has to do with the severe restriction on sets of orthologs and paralogs we included in our data - we required that each rosid manifest at least one copy of the gene in a common syntenic context when compared to the other rosids, and the same for paralogs within each genome. This requirement attempted to isolate the process of fractionation from other processes of gene family dynamics, such as gene movement and gene family expansion, by focusing on the "natural experiment" created by the core eudicot triplication, whereby each species started with three copies of the same gene in the three identical syntenic contexts, and each then retained either one, two or three of these copies over time. A working assumption was that fractionation would be the dominant process affecting these particular sets of genes. The requirement, however, reduced the total number of "homology sets" considered to about a third of the number of genes in the extant genomes. Do our results therefore pertain only to genes that are syntenically conserved and comprise the "stable genome" for functional reasons, or are they also valid for some additional class of paralog sets, some of whose elements are not detectible using syntenic criteria? The third and final problem is whether the results we traced on high-level functional categories indicate general tendencies within these categories, or simply reflect the preponderance of some subcategories among many others with diverse, and perhaps contrary fractionation and positional stability behaviour.

In this paper we address these three concerns. First, we replicate our original studies on two other groups of plants, one consisting of three lamiid asterid species: tomato (*Solanum lycopersicum*) [12], humped bladderwort (*Utricularia gibba*) [13], and monkey flower (*Mimulus guttatus*) [14], the other, four Poaceae monocot species: rice (*Oryza saliva*) [15], foxtail millet (*Setaria italica*) [16], sorghum (*Sorghum bicolor*) [17], and purple false brome (*Brachypodium distachyon*) [18], thus compiling the first pan-angiosperm study focused on gene fractionation patterns. The asterids have all individually undergone further WGD since the core eudicot whole genome triplication they share with the rosids. Nevertheless, the principles underlying functionally-influenced fractionation can be assumed to hold just as well after several WGD as after one. The divergence of the asterids from the rosids seems to have occurred within five or ten million years after the triplication event they shared approximately 120 million years ago [19], so that 90 - 95% of their evolution, including much of their fractionation, took place independently in the two groups. The monocot species in our study all share two or three WGD independent of the all polyploidy events in the eudicots. We will show that the independent fractionation patterns of all three diverse groups of angiosperms are highly parallel, following the same patterns of fractionation based on gene functional classes.

Second, we investigate the hypothesis that our results only apply to a non-mobile core of genes that are detected by the syntenic context they share with orthologs in all the genomes in the group. We relax somewhat the requirement that all the extant genomes must contain at least one syntenically validated descendant of the ancestral gene. Even though the additional cohort of genes, part of the more "mobile" genome that is prone to translocation in a genome, is less numerous and thus less conducive to statistical significance of the results, we find that they continue to support the tendencies found for the functionally "stabilized" part of the genome. We demonstrate that the homology sets we study are enriched for some categories and depleted for others, in comparison with random samples of genes from the entire extant genomes, but hypothesize that this has more to do with lineage-specific expansion of gene families, rather than fractionation dynamics.

Finally, we search for tendencies within subcategories of some of the large functional categories. We find that the negative slope within the catalytic activity category reflects in large measure the consistent behavior across certain classes of enzymes. We resolve the apparent conflict between the trends for the high-level terms "organelle" and "organelle part" seen in Figure 1 by de-convoluting lower level terms contained therein.

## Methods and data

We compared the retention of homologs in six core eudicot species, namely three rosids: peach, cacao and grape, and three asterids: tomato, *Utricularia *and *Mimulus*, and four monocot species: rice, *Setaria*, sorghum and *Brachypodium*, forming three independent data sets.

**Figure 2:**
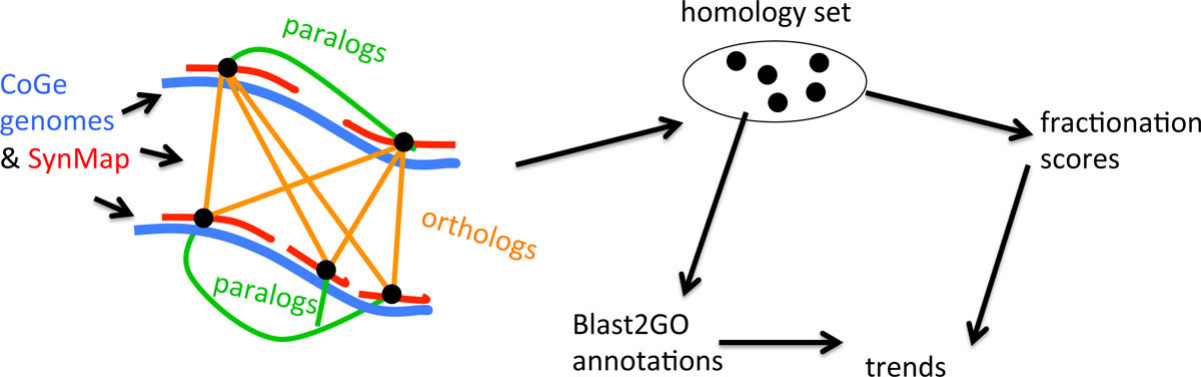
**Pipeline detects functional determinants of fractionation rate**. Blue lines represent chromosomes in two genomes; red lines all represent similar syntenic contexts.

The data preparation in our approach, illustrated in Figure 2, starts with applying the SynMap program in the CoGe platform [20,21] to selected pairs of genomes stored on the CoGe site. This produces synteny blocks of genes (five or more, in the present work) likely to be orthologs because they have high sequence similarity and are in the same syntenic context. This includes paralogous genes syntenically mapping to the same ortholog(s). Additional syntenic paralogs derived from polyploidy can be detected through SynMap self-comparisons of genomes. All the genes sharing orthologies and paralogies thus detected, among all the species in each data set are then grouped together yielding "homology sets" representing ancestral pre-WGD genes [22].

The homology sets were first examined to see whether they contained at least one gene from each species in the group. In the first analysis, all sets with no gene in any of these species were excluded from the analysis. (In a later analysis, described below, other homology sets were used.) The remaining sets were classified according to the number of species in which there was more than one copy, so that in the three-species comparisons, the sets could be classified as 0, 1, 2, or 3, and in the four-species set a score of 4 was also possible. We call this number the *fractionation score*.

For each homology set, each of its genes was annotated by submitting it to Blast2GO [23]. Then all the annotations from all the genes in this set were considered as annotations for the set as a whole. No account was taken of the multiplicity of "hits" of a single annotation within the set. Of course, for every annotation, each of the higher-level terms of each hit was also counted as an annotation.

Among all the homology sets we constructed, approximately 90% hit at least one GO term, resulting in 10,688 monocot, 6360 rosid and 4638 asterid homology sets for further analysis.

The GO terms are divided at the highest level into "Biological Process", "Molecular Function" and "Cellular Component" and there are a further 67 terms at the next level, which we call "high-level terms". Homology sets with large fractionation scores, i.e., which contain more than one paralog in all or most genomes, tend to have a higher total number of annotations, simply by virtue of having a larger number of genes. This leads to the artifactual observation that almost all functional categories are more favored by homology sets with high fractionation scores. To correct for this bias, we use a normalized proportion of hits for each term for each fractionation score. This is calculated as the number of hits of the term over all homology sets with this fractionation score, divided by the total number of sets with hits for any terms within the appropriate highest-level term. Thus, if "organelle" received 100 hits in all homology sets with fractionation score 3, and if the number of sets hitting any "Cellular Component" term is 300, the normalized "proportion" is 33.3%.

These normalized proportions could then be plotted against fractionation score as in Figure 1. By considering every combination of homology set and functional category as a data point with X-coordinate its fractionation score and its Y-coordinate 1 or 0, depending on whether the homology set was a hit (1) or not (0) for that category, we could then calculate a regression score for the functional category. In Figure 1, the functional categories with significant negative slopes are black and those with significant positive score are red or orange.

## Trends for high-level categories

**Figure 3:**
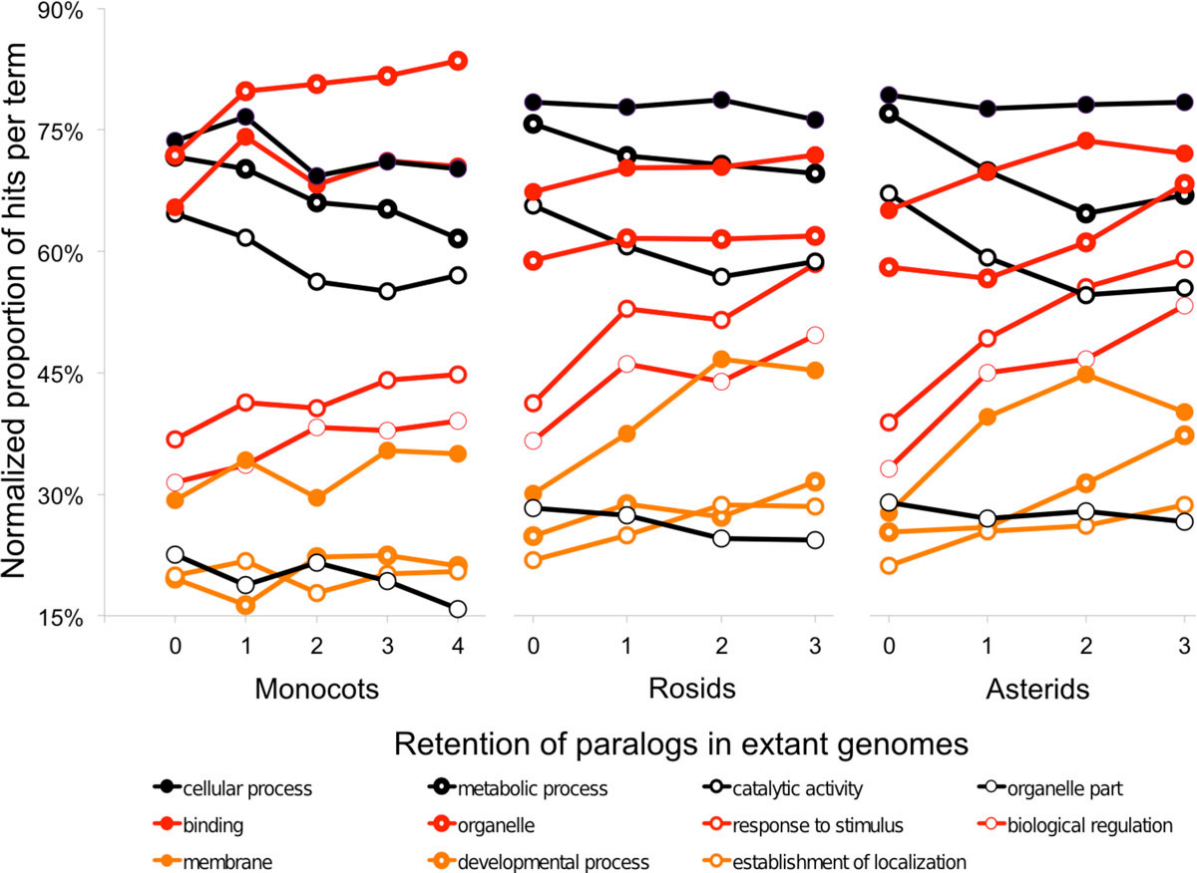
**Fractionation patterns of functional groups in three groups of flowering plants**. Axes as in Figure 1, except that monocots include four genomes, where rosids and asterids include three each.

In comparing the fractionation patterns of the three groups of species, we looked for any trends that were statistically significant in at least one of the three (preferably all three) and with similar slopes in all three. Of the 67 high-level terms in the GO hierarchy, eleven that satisfied these conditions are illustrated in Figure 3. Another 19 terms also satisfied the conditions but involved numbers of homology sets too small to be informative on the figure. Only three terms, or 5%, were significant in opposite directions in two groups of species.

Thus, the three patterns are surprisingly parallel. Recall that the monocot ancestor and its WGD occurred in a completely different part of the flowering plant phylogeny from the eudicots. And though the two groups of core eudicots descend from the same triplication event, the common ancestor of the asterid lamiids evolved for a lengthy period before radiating into the present-day families. Similarly for the common ancestor of peach and cacao, although grape belongs to an early branching rosid order.

The Gene Balance Hypothesis predicts that genes involved in multi-unit protein complexes, or genes involved in cascades in which the downstream genes are involved in multi-unit protein complexes, are more likely to be retained after whole genome duplication. This prediction can in turn predict what GO terms are expected to be fractionation resistant or fractionation prone. The predicted terms are largely the same as the significant in Figure 3, especially "binding", "biological regulation", "response to stimulus" and "developmental process".

Of the significant terms, two stand out as being fractionation prone: "metabolic process" and "catalytic activity". These two terms describe processes or functions that, as traditionally conceived, involve the interactions of single enzymes with substrates, coenzymes and cofactors that are not themselves proteins. This is in contrast with such processes as gene regulation, which explicitly involve the stoichiometry of two or more distinct proteins. On this basis, the Gene Balance Hypothesis would predict that "metabolic process" and "catalytic activity" be fractionation prone. There is much recent enthusiasm, however, for protein complexes in metabolic and other catalytic reactions, for metabolons [24], protein heteromers and other potential sources of abundance constraints among different proteins, so such predictions might have to be attenuated according to how widespread and how constraining these structures turn out to be (cf [25,26] as examples of more moderate opinions).

## The "mobile" genome

All homology sets involved in the above analysis contain at least one gene from each species To test whether this requirement biases the analysis towards genes with some special functional properties, we also carried out our analysis on homology sets where no ortholog was detected in one of the genomes. The absence of this gene from a synteny block does not necessarily mean that it is absent from the genome; it may have moved to some other location on the same or different chromosome.

**Figure 4:**
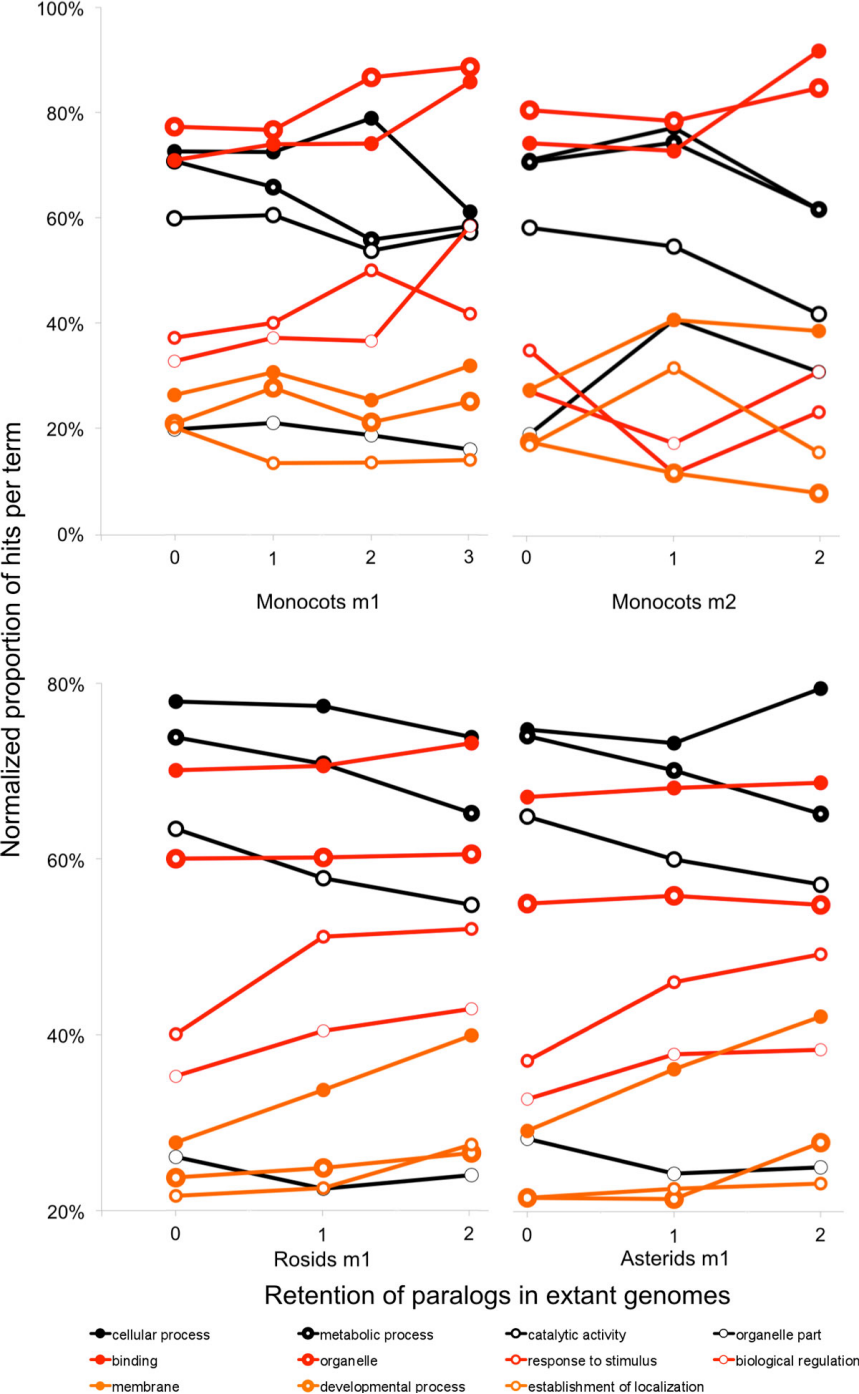
**Fractionation for homology sets containing no gene for one species (m1) or two species (m2)**.

In the case of the monocots, we also constructed a data set where genes were absent from two of the four species, one from the Panicoideae (sorghum or foxtail millet) and one from the so-called BEP clade (rice or brachypodium). These data sets are smaller than the ones composed of full homology sets that we have been analyzing, containing 2532 monocot sets missing one gene, 833 missing two, 4638 rosid sets and 5510 asterids, but their fractionation patterns are remarkably similar as depicted in Figure 4. This analysis offers no support for the idea that the stable and mobile parts of the genome fractionate in different ways, though it only pertains to a restricted portion of the mobile genome.

## Comparison of "stable" genome and the general gene complement

**Figure 5:**
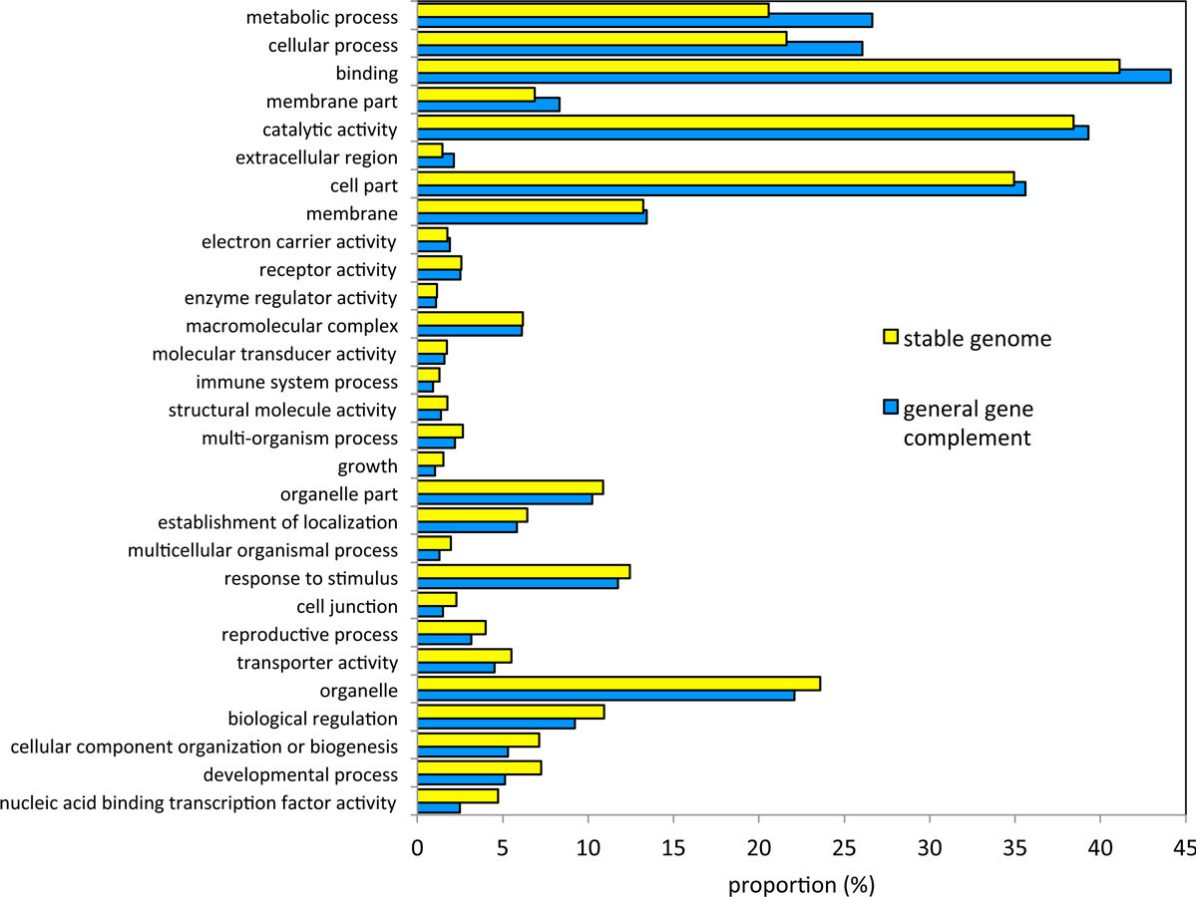
**Comparison of homology sets with sample of three extant genomes, according to hits in functional categories**.

Comparison between our rosid homology sets, reflecting genes syntenically conserved from the original polyploid, and a random set of 9000 genes sampled from the three rosid genomes, unconstrained by homology and syntenic context, shows differences with respect to several GO terms (Figure 5) Terms such as "developmental process", "reproductive process", "biological regulation", "response to stimulus", "establishment of localization" and "cellular component organization or biogenesis" consist of much higher proportion of our homology sets than of the sampled extant genomes. Most of these, though not "response to stimulus", are consistent with the idea of stable gene complement in these areas of reproduction and development, less focused on the interaction of the cell and its external environment. In contrast, the enrichment of the extant genomes with respect to "externally oriented" membrane terms, extracellular region, immune system, metabolic and catalytic terms reflect high rates of gene family expansion, such as by tandem duplication, and other innovations in these categories. Fractionation of ancient syntenic paralogs would thus not play a large role in these differences.

## Trends for more specific categories

Finally, we search for tendencies within subcategories of some of the large functional categories. We find that the negative slope within the catalytic activity category reflects in large measure the consistent behaviour, across all three data sets, of isomerase, hydrolase, and some transferase subcategories, while other transferases notably the kinases and those transferring phosphorus-containing groups, as well as several subcategories of oxidoreductase activity are actually fractionation resistant. The details:

• Oxidoreductase: No overall tendency, but 5 of 88 terms one level lower are fractionation resistant, significantly so in at least one of the data sets:.

- monooxygenase activity

- oxidoreductase activity, acting on diphenols and related substances as donors

- oxidoreductase activity, acting on X-H and Y-H to form an X-Y bond

- arsenate reductase activity

- oxidoreductase activity, acting on the aldehyde or oxo group of donors

• Transferase: No overall tendency,

- (prone) transferase activity, transferring one-carbon groups

- (prone) methyltransferase activity

- (resistant) transferase activity, transferring phosphorus-containing groups

- (resistant) kinase activity

- (prone) nucleotidyltransferase activity

• Hydrolase: Fractionation prone on the general enzyme class level and 2 of 21 lower terms. "Peptidase activity" is fractionation prone (in conflict with [8]).

- (prone) hydrolase activity

- (prone) peptidase activity

- (prone) hydrolase activity, acting on carbon-nitrogen (but not peptide) bonds

• Isomerase: Fractionation prone on the general enzyme class level and 2 of 14 lower terms.

- (prone) isomerase activity

- (prone) cis-trans isomerase activity

- (prone) intramolecular transferase activity

• Lyase: Has conflicting slopes among the three data sets at the general enzyme class level. No fractionation prone or resistance from 13 lower terms.

• Ligase: No tendency detected

The apparent conflict between the trends for "organelle" and "organelle part" turns out to be due to be the 95% concentration of the latter in chloroplast terms, where only 30% of the former are so annotated.

## Connection between fractionation rates and and paralog retention patterns

In our previous study [4] we modeled the duplicate and triplicated data depicted in Table 1 in terms of a single rate of loss of *p *for the period between hexaploidization and speciation and individual loss rates qi,i= 1, ⋯ ,6 for each of the six species starting from an assumed common speciation event. (This assumption is a major biological simplification with, however, no numerical consequences for this particular model.)

**Table 1:** Numbers of triples and pairs after fractionation in six rosids.

	frequencies of gene family sizes					
**size**	**peach**	**cacao**	**grape**	**castor bean**	**strawberry**	**papaya**

2	1484	1111	945	851	606	474
3	256	172	150	119	57	34

Data from [4]

In the model, the probability that

1. an original paralogy triplet would survive intact is $\frac{{\left(1-p\right)}^{3}}{1-p^{3}}\frac{{\left(1-q_{i}\right)}^{3}}{1-q_{i}^{3}}$.

2. an original triplet would manifest as a pair is $\frac{{\left(1-p\right)}^{3}}{1-p^{3}}\frac{3q_{i}{\left(1-q_{i}\right)}^{3}}{1-q_{i}^{3}}+\frac{3p{\left(1-p\right)}^{3}}{1-p^{3}}\frac{{\left(1-q_{i}\right)}^{2}}{1-q_{i}^{2}}$, and

3. an original triplet would be reduced to a single copy is $\frac{{\left(1-p\right)}^{3}}{1-p^{3}}\frac{3q_{i}^{2}\left(1-q_{i}\right)}{1-q_{i}^{3}}+\frac{3p{\left(1-p\right)}^{2}}{1-p^{3}}\frac{2q_{i}\left(1-q_{i}\right)}{1-q_{i}^{2}}+\frac{3p^{2}\left(1-p\right)}{1-p^{3}}$.

Because this model is inspired by concepts of the stable genome, the frequency of single-copy genes, which tend to be part of the mobile genome, are not part of the input data in Table 1, and so the original number of triplicates in the stable moiety, before fractionation, has to be inferred statistically.

It was suggested in [4], and motivated in part the functional analysis in that research, that a better fit of the model to the data would be obtained by allowing for two or more gene classes with different rates. Thus we have modified the model by dividing the genes into two classes, fractionation-prone and fractionation-resistant in proportions *θ *and 1 − *θ*, with a parameter *α *linking the rates for the two classes:

$$\begin{array}{c} \text{class }\theta \text{ genes}:p,q_{i},i=\text{1},\cdots \;,\text{6}\\ \text{class 1}-\theta \text{ genes}:\alpha p,\alpha q_{i},i=\text{1},\cdots \;,\text{6} \end{array}$$

**Figure 6:**
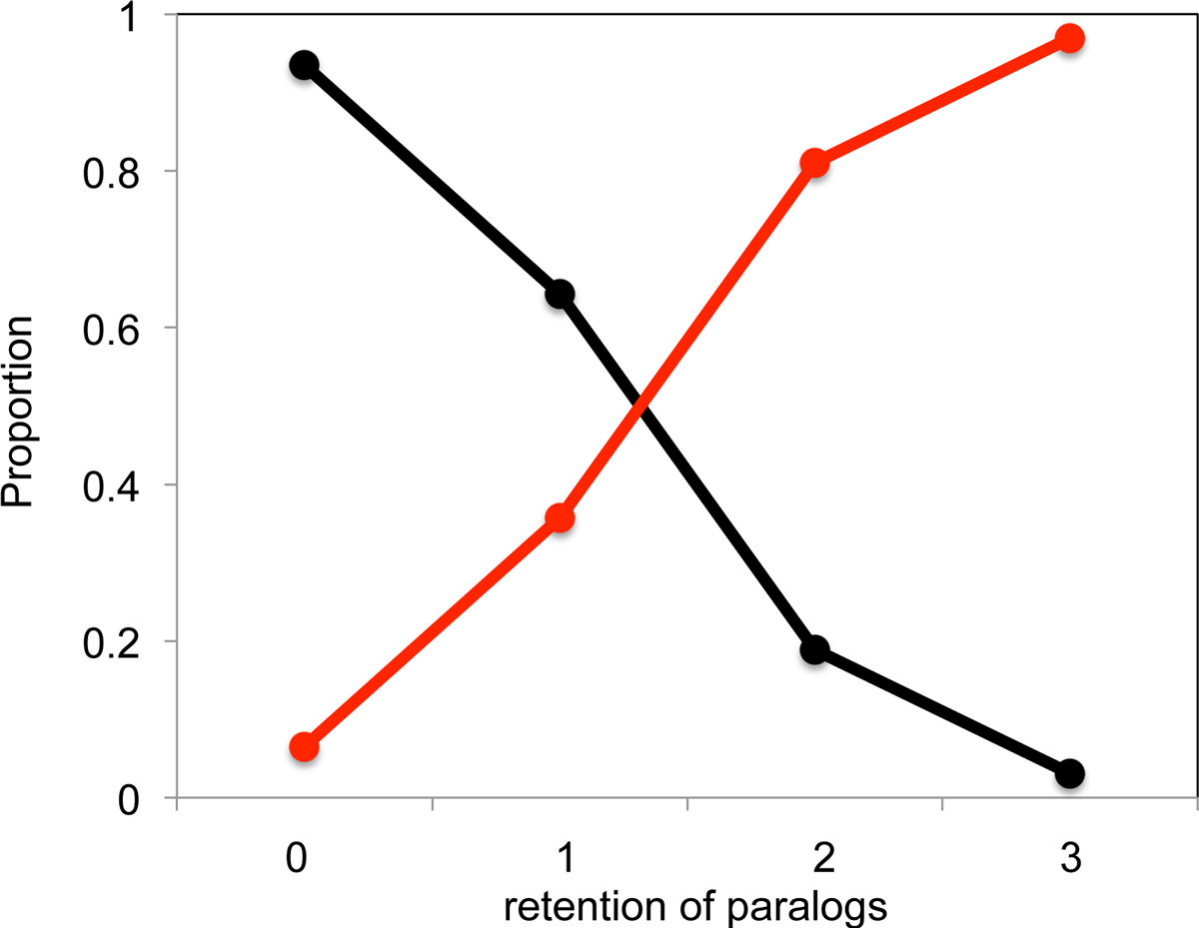
**Predicted paralog retention patterns in rosids for model with two gene classes**. The black line represents the fractionation-prone gene population and the red line represents the fractionation-resistant population.

Based on the data in Table 1, with an initial number of pre-triplication genes of 7500, the maximum likelihood fit produced two populations with 75 % and 25 % of the genes, respectively, and with a relative rate parameter *α *= 0.60. For larger initial numbers of genes, only *p *varied somewhat to compensate, while the estimates of the *q_i_, θ *and *α *were stable. From these estimates, we could predict the number of genes in each class using the same x-axis as Figures 1 and 3, based on *p *and *q_i _*for the peach, cacao and grape genomes, and plot the relative proportions of the two gene populations in each class, in Figure 6.

We refrained from trying to model the annotation process, so the results in Figure 6 are more contrastive of the two population of genes than the real data. Nevertheless, it is encouraging that the empirical data in Table 1 contain the signal of two classes of genes with different fractionation rates, reminiscent of the patterns in Figures 1 and 3.

## Conclusion

Building on our previous results on the functional determinants of fractionation of rosid genomes, we showed that the independent fractionation patterns of rosids, asterids and grasses are highly parallel. The same functional categories of genes are preferentially fractionation prone or resistant.

That the asterids demonstrate the same patterns as the rosids and monocots confirm that there is a general trend for paralogs to be fractionation prone or resistant, as influenced by their functional categories, despite the additional WGD events in the asterid lineages. This may be attenuated by changing fractionation patterns for some gene copies from one WGD to the next, as reported recently [27].

Additionally, we tested whether these patterns of fractionation were consistent between the stable and mobile portions of the genome. Even though the additional cohort of genes, part of the more "mobile" genome, is less numerous and thus less conducive to statistical significance of the results, we find that it supports most of the tendencies found for the functionally "stabilized" part of the genome. Our analyses support that there is no fundamental difference between the strictly stable and partly mobile parts of the genome. Rather the same functional categories influence the fractionation fate of genes. Nevertheless, we also demonstrate that the homology sets we study are enriched for some categories and depleted for others, in comparison with random samples of genes from the entire extant genomes. This likely has more to do with lineage-specific expansion of gene families, rather than fractionation dynamics.

Perhaps the most important improvement to be envisaged for our method would be to ensure that the homology sets contain only paralogs created by the initial WGD event. Tandem duplicates or other duplicates produced more recently than the WGD may constitute a major proportion of all the duplicates in a genome. Although SynMap normally excludes tandem duplicates, some of them undoubtedly remain and may be reintroduced when we combine pairwise genome results to form homology sets. Indeed, for future work, it would be worthwhile to contrast the paralog loss behaviour from WGD fractionation with that from other sources of duplicates. This approach was pioneered in [28] for the *Arabidopsis *genome, with results very similar to our approach, suggesting our comparative approach might bolster these findings.

Our use of general GO terms could conceivably be improved by using a more focused gene ontology database such as Plant Slim developed by the Arabidopsis Information Resource. Indeed, preliminary tests show that our results on high-level terms could be sharpened using this resource, but unfortunately there is relatively little annotation at present using lower level terms, so this avenue is limited for the moment.

It may advance the understanding of functional associations of fractionation to compare the correlations among functional categories enriched for fractionation-prone genes in contrast to the correlations among these categories for fractionation-resistant genes. It is not a methodological flaw that many categories are correlated among themselves -- indeed it opens up the opportunity to compare changes in these correlations for genes with differing fractionation scores.

## Competing interests

The authors declare that they have no competing interests.

## Authors' contributions

EC and DS planned the research for this article. EC carried out most of the research and writing. EC, DS, EL, VAA, LC-P and HT participated in the interpretation of the results. CZ did the preparation of the genomic data and helped in the computer analysis. AB did the probability modeling and calculations. CFBAN assisted in the data analysis and preparation of the manuscript. All authors participated in writing. All authors read and approved the manuscript.

## Declarations

Publication of this article was supported by the Canada Research Chair in Mathematical Genomics.

This article has been published as part of BMC Bioinformatics Volume 14 Supplement 15, 2013: Proceedings from the Eleventh Annual Research in Computational Molecular Biology (RECOMB) Satellite Workshop on Comparative Genomics. The full contents of the supplement are available online at http://www.biomedcentral.com/bmcbioinformatics/supplements/14/S15.
